# Convergently Emergent: Ecological and Enactive Approaches to the Texture of Agency

**DOI:** 10.3389/fpsyg.2020.01982

**Published:** 2020-08-07

**Authors:** Marek McGann

**Affiliations:** Department of Psychology, Mary Immaculate College, University of Limerick, Limerick, Ireland

**Keywords:** agency, enaction, ecological psychology, emergence, scales

## Abstract

Enactive and ecological approaches to cognitive science both claim a “mutuality” between agents and their environments – that they have a complementary nature and should be addressed as a single whole system. Despite this apparent agreement, each offers criticisms of the other on precisely this point – enactivists claiming that ecological psychologists over-emphasize the environment, while the complementary criticism, of agent-centered constructivism, is leveled by ecological psychologists at enactivists. In this paper I suggest that underlying the confusion between the two approaches is the complexity of agency, which comes in different forms, at different scales or levels of analysis. Cognitive science has not theorized the relationship between these different forms in a sufficiently disciplined manner, and a task therefore remains of finding a way to map the complex territory of agency.

## Mutual Mutuality

Both ecological and enactive approaches to cognitive science begin with a recognition of a mutuality, or reciprocity, between an agent and its environment. The agent-environment system must be studied as a whole. The environment must be defined in terms of the agent, the world as it is engaged with and experienced by the agent; not an abstract and neutral domain about which the agent must reason. In a complementary manner, the agent must be understood not, for instance, as an abstract processor of information or logic engine, but an embodied being connected with and in continuous interaction with the world around it.

Despite these apparently shared foundations, the two research communities have had surprisingly little to say to one another. While they agree on some of the premises, both approaches have quite distinct perspectives and emphases, which seems to have led each to approach this mutuality between agent and environment from opposite directions. As would be predicted from either perspective, the particular orientation taken significantly affects the form of engagement between the research community and their subject matter. In this case, while both camps argue that a full circle of mutual influence and dependence must be drawn between the two aspects of a cognitive system (considered here as the agent-environment whole), just how you perceive that mutual relation will depend on where on the circumference you decide to first place the pen.

In what follows I argue that enactivists and ecological psychologists accuse each other of complementary violations of this shared principle of mutualism. Enactivists (Varela et al., 1991) claim that ecological psychologists are guilty of stipulating specific structure in the world prior to the agent and thus attempting to build an account of their relationship from just the environmental aspects in question. Ecological psychologists, meanwhile, argue that enactivists are guilty of requiring that structure to be stipulated *post-hoc* by the agent (Fultot et al., 2016) thus attempting to get the agent to do all of the meaningful work. I argue that this complementarity of criticism is driven by the ways in which we have addressed the question of different forms of agency to date, particularly with regards to the interaction between those different forms at various scales. I argue that both approaches share an emergentist stance that can help move us forward on the issue, that this stance raises other important points of consideration regarding the kinds of agency that exist, and the relationships between them.

## Complementary Mutualities

Though parallels had been noted from the beginning, Chemero (2009) was perhaps the first to suggest a potential complementarity between ecological and enactive approaches to cognitive science. Both take what might be called a “radical embodied” perspective, one which denies the need of representational states underlying cognition. Their shared mutualist framing of the question means they both place an emphasis on the embodiment of the agent and the dynamics of its interaction with its environment. Chemero discussed the apparent complementarity of ecological psychologists’ examination of the environment and its structures that empower the engagement between it and the agent, while enactivists have explored the details of how the structure and dynamics of the agent affect that same relationship.

### Ecological Psychologists Focusing on What You Are in, Rather Than What Is in You

Ecological psychologists have addressed themselves to the question of how the environment supports perception and action by embodied agents. Their work is some of the most robust, precise, and successful that has been conducted in psychological science. Researchers have identified a range of ways in which structure in ambient arrays of energy or chemistry can be coupled with by an animal (or other embodied agent) to engage in some form of goal-directed action.

While some approaches see the abundance of detail in the environment as a problem that must be overcome to avoid the agent being overwhelmed, for ecological psychologists it is in fact a blissful wealth of specificity that means we can get an awful lot of work done without needing complex inferential processes (Chemero, 2009; Fultot et al., 2016). Perception and action are two aspects of the same phenomenon (Turvey et al., 1981; Gibson, 1986) and perceptual skill is not about piecing sensory stimuli together to form a complex mental structure but more of a matter of making better and better discriminations regarding the fine-grained details of the environment within which we are acting (Gibson and Gibson, 1955).

The various aspects of the environment that afford such effective action have been examined in some detail by ecological researchers and this has from the outset included a recognition of the importance of the body of the agent, or animal^1^. If perception is relative to action, then actions of which my body is capable matter to what I will perceive.

But there has also long been some uncertainty about how that animal should be considered, and it is on this issue that apparent conflict between the otherwise consonant approaches of enactive and ecological cognitive science first arose. In their initial statement of the enactive approach Varela et al. (1991, p. 202–203) explicitly take issue with Gibson’s ecological work, arguing that it attempts to build an account of the relationship between the agent and the environment entirely from the side of the environment, and thus appears to violate the principle of mutuality. Though they note in a footnote (Varela et al., 1991, p. 274–275, 38n) that where the relationship is characterized as emergent within the animal-environment system (as is argued by Turvey et al., 1981 for instance) then some of this tension is dissolved. The heavy (though not exclusive) emphasis on the environmental rather than animal-environmental structures in the relationship remained a point of contention. While Varela et al.’s comments did not prompt an extensive dialog between ecological and enactive researchers, questions about the specification of the embodied agent in ecological psychology have also been raised by those working within the ecological approach.

Stoffregan and Bardy (2001) and Stoffregen et al. (2017) point out that Gibson, and almost all of those who have extended his work, have maintained a sharp distinction between structure in the environment (in particular in the arrays of ambient energy), and structure within the organism. They make this point with reference to perceptual modalities, arguing that Gibson (and subsequent ecological work) generally maintains unexamined traditional distinctions between systems – the visual, haptic, auditory, and so on, each coupled to structure in a particular array of ambient energy to which they are sensitive. Stoffregen and Bardy (2001) argue that these are inappropriate distinctions, made by scientists for the sake of organizing their work (and partly just following traditional categories descended from Aristotle’s) but not by perceiving agents under normal circumstances. Gibson himself noted that the visual system includes not just the eyes and brain but also, for instance, the trunk and legs of an animal playing a role in orienting the organism and coordinating its movements with the structures in ambient light. Therefore what is involved in visual perception involves the body of the organism, not just the structures of those rays of ambient light. In a sense this is part of Gibson’s recognition of the importance of embodiment, and is present in foundations of his ecological approach, but its implications remain insufficiently appreciated. Stoffregen and Bardy (2001) argue that the role of the body in coupling of action to structure in the ambient arrays means that it is not possible give an account of the coupling on the basis of the ambient arrays alone. There can be no case of “pure” vision in terms, for instance, of optics, as the body, and at least kinaesthetic and vestibular systems are always also implicated. The structures of energy in question are thus never purely optical, but complex or “compound.”

Structure in ambient arrays is generally taken to reveal invariants in dynamics that can support coordination between an organism and its environment. The most frequently discussed is structure in the rays of ambient light, but Gibson explicitly notes the existence of compound (Gibson, 1986, p. 141) higher order invariants in complex arrays of multiple forms of energy. Stoffregen and Bardy (2001) simply point out that a moment’s consideration will tell us that such compound invariants are the norm, rather than the exception. In the normal case, the world is perceived not as a set of combined modal components but a complex perceptual whole, though one which might, as necessary, be interrogated according to modal characteristics. There are not separable perceptual systems, sensitive to distinct ambient arrays, which are combined to create some complex percept, but rather a single complex perceptual system capable of coming into coordination with rich structure in a complex “global array” (Stoffregen and Bardy, 2001).

The surprising criticism leveled by Stoffregen and Bardy (2001) at Gibsonian ecological psychology is, therefore, that it does not take the embodiment of the point of observation seriously enough. A strange criticism to see made of the scientific community who can be argued to have been doing embodied, and even “radically embodied” (Chemero, 2009) cognitive science longer than anyone else (Fultot et al., 2016). But I find Stoffregen and Bardy (2001) and Stoffregen et al. (2017) arguments compelling (you could say I’m predisposed to, though McGann (2010) was written in shameful ignorance of their work). Perceptual systems are entangled in the body and isolating individual modalities in experience is more an achievement of disciplined phenomenology and scientific practice than the default state of affairs. The body does not work to put together separate sets of ecological information from distinct arrays, but creates structures of complex ecological information by being the point of entanglement and inter-relation between those distinct arrays. The global array comes into existence with the agent, and *only* with the agent, and to properly account for the invariants that support coordination between agent and environment requires that agent to be specified to an extent that Stoffregen and Bardy (2001) and Stoffregen et al. (2017) claim is rare in ecological research.

The mutuality tenet would also suggest that this limited account of the agent also underlies an ambiguity concerning Gibson’s description of the environment discussed by Baggs and Chemero (2020). Gibson in *The Ecological Approach to Visual Perception*, distinguishes between the physical world (as studied, for instance, by physicists), which is not animal-relative, and the environment, which is. Baggs and Chemero argue that Gibson is ambiguous in his descriptions of this second sense. Sometimes Gibson is referring to a generic environment shared by all animals with similar embodiment (for instance, members of the same species), which Baggs and Chemero suggest calling the “habitat.” And sometimes he uses the phrase as unique to individual organisms with their particular learning histories, which Baggs and Chemero term “umwelt.” They suggest that the continuation of this ambiguity in subsequent work by others helps understand a number of confusions or controversies that continue within the ecological literature to date.

Of course, from a mutualist perspective two separate considerations of the environment imply distinguishable conceptions of the complementary agent. Ambiguity in discussion of the environment implies a concomitant ambiguity in specification of the agent in question. Were differences and varieties of agent and agency more explicitly theorized, it would seem less likely that any ambiguity in discussion of the environment could have been maintained, particularly for so long without people being keenly aware of the issue.

And so we return to Chemero (2009) suggestion that an enactive approach offers a useful complement to the ecological flavor of radically embodied cognitive science, partly as he identified this need to address the individuality, rather than generality, of relationships between specific concrete agents and their environments. As ecological psychologists appear to have stumbled over a complexity of agency in the complex system of animals interacting with their environments, enactivists, who can be seen to have approached the complementarity from the other direction, have been struggling with it so much that they have barely got around to looking at the environment at all. This focus on agency by enactivists is to the point that Fultot et al. (2016) argue that enactivists have given too much account of the agent’s role in the interaction that it looks like they are offering little more than a warmed over serving of mental representations, a mental constructivism by which the agent “brings forth a world” from a meaningless soup of ill-specified environment. Some approaches, it would seem, are more mutual than others.

### Enactivists Focusing on What You Are

In contrast to ecological psychologists’ heavy emphasis on questions of the environment, enactivists have spent 30 years trying to get to grips with the fine-grained details of agency – how it arises, how it operates, in what forms it can be found.

Enactivists put forward a naturalistic account of value, which in the abstract is a system of processes that, together, continue to produce that very system, essentially becoming an enabling condition for its own existence. This is done under conditions of precarity (Di Paolo, 2009) which is to say that without the self-supporting organization, the system would tend to run down or disintegrate. Though self-sustaining, it is considered more a continuation of a trajectory or dialectic than a maintenance of a fixed or rigid set of relations or variables (Di Paolo, 2018). Understanding agency means understanding both the values that animate it, and the constraints (bodily, worldly, and various things in between) that underpin it.

We should not be surprised that as ecological psychologists have found structures in the environment at various scales and degrees of complexity, so enactivists have identified agency as having a variety of forms and scales too. I will not attempt a systematic enumeration here, but it is worth noting two relatively separable strands within the enactive literature: one exploring issues of life, skill, and agency within the domain of sensorimotor activity (Maturana and Varela, 1987; Varela, 1997; Weber and Varela, 2002; Di Paolo, 2005; Thompson, 2005; Buhrmann et al., 2013; Beaton, 2016; Di Paolo et al., 2017; Froese and González-Grandón, 2020) and a second exploring the perhaps more complicated and differentiated world of social and cultural dimensions of the same, in which agency inheres not necessarily within individual bodies, but also across them (De Jaegher and Di Paolo, 2007; De Jaegher and Froese, 2009; McGann and De Jaegher, 2009; Torrance and Froese, 2011; Kyselo, 2014; Cuffari et al., 2015; Cummins, 2018; Di Paolo et al., 2018). We should also note that there is a great deal of complexity being teased apart within each of these strands.

Identifying values that are not inherent in individual bodies, but which encompass more than one is both necessary (how else can we come to terms with basic multicellarity, for instance), but also subversive. Introduced first in terms of participatory sense-making by De Jaegher and Di Paolo (2007) it is a recognition that the values animating and organizing actions cannot be fully described or explained by the theorizing of the individual agent.

Several significant works in the field are perhaps best understood as attempts to braid or knit these two strands of enactive research together, finding ways in which the different forms of complex, particularly human, agency, can be understood as consistent and inter-related. Cummins (2018) for instance, explores in detail the ways in which different domains of human existence feed back on one another, highlighting that human beings are multiply animated, committed to and engaged with a complex of different processes which both motivate, enable, and constrain our activities in complex and often conflicting ways. Cummins begins with an examination of the universal, but little studied phenomenon of joint speech, when more than one person says the same thing at the same time. It is a striking feat of inter-personal coordination that is both achieved with apparent ease and deployed in circumstances of significant importance to identity and collective action. Seen across cultures in situations ranging from prayer to protest, education to sports fandom, Cummins examines a plethora of examples to tease out the implications regarding different forms of subjectivity and agency that encompass much more than individual organisms. He highlights how these different forms of agency arise not just from biological systems, but at confluences between biological, moral, civic, and other domains of activity, and must be understood in those terms - it is a sensorimotor skill that, in its naturally occurring enactment, inescapably highlights the need to understand it within moral and civic reference frames.

Though we are individuals, we are rarely *just* individuals, but rather simultaneously enacting a number of different forms of agency, and bringing them into greater or less degrees of coordination. Some of the values which animate human bodies are not inherent wholly within those bodies.

Di Paolo et al. (2018) *Linguistic Bodies* involves a slow, cautious examination of the ways in which bodies engage in sense-making, an adaptive process of skilful coping by the agent with the environment in which it finds itself. Enactivist work has examined ways in which the materiality and history of an agent affect the dynamics of its agency; the body, environment, and activity that combines both are in a constant process of pull and press that has emergent dynamics at multiple scales. The individual agent (with its particular body, history, and skills) matters, and must be understood through the ways it is engaged in several different ways with the world around it. While we might identify a number of different domains of activity within which a particular agent is embedded – sensorimotor, discursive, social, cultural, others – all of these domains are entangled through the body of the agent and Di Paolo et al. (2018) make a first attempt at mapping those relationships and finding ways to describe and consider them systematically.

There is I think, a real but distant resonance here with the insight of Stoffregen and Bardy (2001) whatever domain of activity in which you as a scientist are interested is tangled up with everything else that the agent does, because the agent’s embodiment is necessarily embedded in it all. What we as scientists perceive as distinct domains of activity might be addressable in that way for the purposes of conducting particular forms of research, but are also entangled and inter-related with one another by the (rather messy and complex) unity of the embodied agent. A mature cognitive science will include means by which such orientation can be done in a systematic manner, in perhaps a similar way that biologists are able to orient themselves within subfields of biochemistry, genomics, morphology, and ecology.

The strong agency-focus of enactive research has allowed a range of detailed aspects of agency and sense-making to be developed, but in that process relatively little has been said by enactivists about the environment with which these various forms of agency are mutual (McGann, 2014). Fultot et al. (2016) take enactivists to task over this imbalance, arguing that this failure to adequately address the environment, and too-heavy focus on the agent, has resulted in a violation of the principle of mutuality and an at least implicit requirement for a constructivist agent – one that constructs its environment rather than encountering it directly and meaningfully. There are several commentaries on the Fultot et al. paper, along with the authors’ response, in the issue it appears, and I will not rehearse the debate here. Suffice it to say that several enactivists (myself among them) acknowledge the criticism, though reject the implied fatal conclusion for our shared principles. Di Paolo (2016, p. 329) points out that while an enactive account of the environment is work that has not been done, it is perhaps most appropriate to say that it is work that has not been done *yet*.

Though it may not be as problematic as some critics suggest, the form and structure of the environment is yet a rather fraught question for enactivists, particularly if the commitment to mutuality between agent and environment is to be maintained, which it must, being one of the principal tenets of the entire approach.

This is not to say that enactive theories of various forms have nothing to say about the environment, any more than ecological psychologists have had nothing to say about the structure or form of the agent. Rather, we might observe in the tendencies toward singular emphasis within each perspective a difficulty in addressing both aspects of the mutuality relation at the same time. Perhaps it is akin to trying to perceive both versions of a Nëcker cube simultaneously – examining one facet as figure seems to push the other into the background. How we should approach the issue of mutuality between environment and agent when there is more than one form of agency to be understood is a question that will need to be approached with care, and in a manner cognisant of the careful balancing act involved.

## Getting Along

### Coping With a Surfeit of Agency

I suggest, then, that the gap between ecological and enactive approaches is substantially a result of the complexity of agency – insufficiently addressed by one, addressed to the point of exclusivity by the other. The result is a remaining lack of clarity of how to conceptualize the more complex relationship between agent and environment that such a recognition entails. We are therefore faced with an interesting challenge; we must find a way to address this complexity that balances key tenets shared by both approaches.

First among these considerations is of course the agent-environment mutuality itself. Having identified it as a characteristic of such systems, we must find a means of describing the agent-environment system that does not impose or require a priority for either one. The agent is not simply caused by the environment, and the environment is not simply constructed by the agent.

Second, we should respect the autonomy of the systems in which we are interested at a given level of description. Both ecological psychology and enactivism hold to a non-reductive account of psychological phenomena – there is no “ground level,” the activity at which explains all else. The physical description of the world cannot adequately capture the existence of particular scales of phenomena which arise in relation to one another rather than in absolute terms. Observed or measured at the wrong grain of resolution and we will miss our phenomena of interest.

This does raise questions about how best to think about the complexity of agency, and bodies as engaged with (and engaged by) values of different levels of analysis. Mind-relevant phenomena occur at a wide variety of scales. From chemical and biochemical processes, to physiological, neural, and biomechanical, to behavioral, eco-behavioral, social, and cultural, there are a host of different perspectives we might take, and within any one of them identify events or processes that relate in meaningful ways to the phenomena of life and mind. This is a rather trite observation to some extent – it is clearly recognized in many ways, not least of which is the existence of several disciplines dedicated to different levels of study, from neuroscience, to psychology, to sociology, and anthropology. The entire field of cognitive science was founded in a recognition that more than one perspective will be necessary to develop a satisfying understanding of mental phenomena. Psychology cannot be a complete science of the mind.

For all of our vaunted inter-disciplinarity, however, cognitive science tends not to do collaborations across disciplines terribly well (Núñez et al., 2019) and collaborations across scales of description would seem to be rarer still (Boden, 2006; Bender et al., 2010; Ignatow, 2014). The question is what kind of framework could be put in place which will allow us to make sense of the relationships between these different scales – recognizing their differences and systematically addressing their interactions. Enactivists have been involved in this effort, though as noted, primarily with regards to the question of agency, with the environment remaining something of a promissory note (Di Paolo, 2016).

If enactivists are correct about the ways in which agency arises (Barandiaran et al., 2009; Di Paolo, 2009; Di Paolo et al., 2018) then there is a consistency in the general form of the dynamics in all cases, being grounded in the dynamics of autonomous networks of processes – different values just aren’t consistently related to the body in the same way. Some of these values are inherent in the bodies of biological agents, but some of these values arise in dynamics that pass through those bodies (De Jaegher and Di Paolo, 2007; Kyselo, 2014; Cummins, 2018). There is therefore no one-size-fits-all account of embodied agency. And yet it remains true that these various forms of agency are all entangled in different ways in living bodies (Kyselo, 2014; Cuffari et al., 2015; Di Paolo et al., 2018) their attributes, powers, and skills. Di Paolo et al. (2018) work in particular examines how multiple forms of agency imply multiple forms of embodiment, though all of them ultimately entangled in the particular concrete form of given agents. Any one body will be animated by multiple such forms of agency, whose relationships we as cognitive scientists must be capable of mapping.

I am simultaneously white, cis-male, middle-class, a father, an academic, writing, and hungry, and my enactment of any and all of these domains of activity is accented by my engagement with the others (to greater and lesser extents). Within the kind of distributed approach to cognitive systems taken by ecological and enactive approaches the individual body is perhaps then to be defined by the particular, unique collection of domains of activity it tangles together. The body is perhaps more a matter of *skein* than *skin*. It should be possible to identify a particular tangle of values and agentive processes that is “me,” but that “me” cannot be exhaustively described within any narrow range of temporal or physical scales, and indeed, it remains to be understood what the specific dimensions of import are (time and space are not likely to be the only ones).

In a somewhat different context, the range of temporal scales of agency has been broached by van Dijk and Withagen (2016). They note the extended, enduring character of human agency, that individual actions are not just punctate events, but are largely manifestations of multiple engagements of many long-duration processes, which can be more or less stable over different timescales. A useful analogy might be the height of the sea – if we pay too much attention to the brief but salient crash of the waves, we can miss the rather important role of the tide. Actions taken, movements made, utterances spoken, are wavefronts borne by tides of mind extending over periods not apparent were we to limit our observations to salient bodily motions (however, skilful). Long timescale cognitive phenomena are occurring right now just as the short ones are. For van Dijk and Withagen (2016) the point was that a radical embodied cognitive science need not be constrained by the traditional distinction between “online” and “offline” cognition, but the implications, I think, are more general.

As we have already noted, this complexity of temporal and physical scales of agency implies a similarly complex complementary environment. Di Paolo et al. (2018) largely address this implicitly – with the environment considered in terms of other agents, and the particular (often messy) details of their specific embodiments. The materiality of those embodiments matters, but the emphasis in their discussion remains on agency, with much work still to do to unpack that implications of the mutuality they nevertheless endorse.

Di Paolo et al.’s work is not alone in recognizing the range of scales of agency. There is also a broader effort by a number of researchers to bridge the apparent gap between sensorimotor and social by drawing in an ecumenical and integrative fashion from both ecological and enactive approaches (Costall, 1995; Rietveld and Kiverstein, 2014; van Dijk and Rietveld, 2017; Bruineberg et al., 2019; van Dijk and Kiverstein, 2020). Clearly, I wholeheartedly agree that we need to find a way to deal coherently with activity in this variety of domains, and that ecological and enactive approaches are valuable resources that should see better integration (I attempted to make my own small contribution to this parsimonious rapprochement myself in the past, McGann, 2014). However, our limited understanding about the relationships between the different kinds of activity has meant that the default approach has been to search for a means of applying the same mode of analysis to all of them, one which emphasizes the autonomy and potency of the individual agent. I have come to suspect, however, that this is in a sense seeking to homogenize these varied environments in order to extend our account of the individual agent across the entire gamut of domains in which that agent is embedded. Such a unifying approach, while laudable in its emphasis on consistency and continuity across scales or levels of analysis, threatens to make us blind to discontinuities and heterogeneity; differences, conflicts, and tensions between the various kinds of value that animate agents’ actions, and therefore the particular ways in which those tensions play a role in organizing and animating the behavior of our systems of interest. It suggests that all of the various scales or domains of activity are ultimately implemented within each individual agent, perhaps by nested systems of the body with dynamics at different temporal scales. This occludes the possibility that some of these forms of agency work across (rather than within) individual biological agents, for instance, meaning they can work sometimes in keeping with, and sometimes in conflict with, other values and forms of agency within which the animal is embedded.

A common mode of explanation in this unifying effort is to appeal to skills of various kinds. We learn to coordinate in all domains of life in which we work through the development of more skills. The problem with such an approach is that this takes the perspective of a given form of agent – the individual biological one – because a skill is by definition a means by which an individual agent comes into increasing coordination with its environment, and these are typically ascribed to individual biological bodies and their relations to the world. If it is the case that the animating values accenting or driving the behavior of a person at any given time may be part of a network of processes that move *through* the individual biological agent, but not be wholly inherent within them, then properly theorizing the environment, and the agent-environment relationship across the different scales of that such processes operate is vital. This is not something I suspect would be controversial for any of the authors I have cited here, but I wonder if the implications for our understanding of agency and bodies have been fully worked through.

If this way of thinking is correct, then it will be important *not* to unify all agencies *within* the body, but to catalog the multiple ways in which the body can be animated, and (crucially) find ways to map the relationships between them. Some values that animate the agent are incorporated in the agent, some incorporate the agent, and we need a coherent and systematic way of moving between the various points of view that enable us to see these different kinds of relationship, without violating the principles of non-reductiveness and mutuality. It is quite likely that grappling with this question will involve a commitment to observing people “in the wild,” so to speak – actually just engaging in a natural history of human behavior to a great and more systematic extent than we have done thus far (Barker, 1968; McGann and Speelman, 2020). But it will also involve engaging in theorizing with the right kind of approach.

### Emergent Media

It is apparently poor form to introduce a problem without also at least hinting at how we might go about solving it. Both camps do, as it happens, share a promising point of departure. The concept of emergence, which is usually deployed in reference to the self-organization of systems under various conditions, appears to fulfill our starting criteria. Both enactive and ecological researchers refer to their approach, or key aspects, as involving “emergence” or “emergent properties” at various times (Turvey et al., 1981; Stoffregen, 2003; Thompson, 2007; Di Paolo et al., 2010; Lobo et al., 2018) typically in order to affirm a non-reductiveness of their account. Emergent properties define their own scale, they are not to be explained away with reference to processes at a single different (usually smaller) scale of description, but must be acknowledged and addressed on their own terms.

An emergentist approach, understood as self-organization of the whole agent-environment system also fulfills the requirement for mutuality, avoiding any stark claims for priority of either facet. It does so, however, at a given level of description, which must be identified if we are to be able to recognize the patterns of interest that are self-organizing.

In looking for patterns not as agents acting in environments, but as cognitive relations without preemptively assigning agency to any particular subset of the system in question, we implicitly distinguish the agent-environment system from a background. That background itself has a set of dynamics associated with it. In essence through our investigations we as scientists create a new agent-environment system, with us coordinating with our target system on the one hand, and an environment within which we are working on the other (a meta-level issue raised for instance, as the topic of second-order cybernetics, Pask, 1996; Von Foerster, 2003). The background is tricky to theorize, but it is not impossible.

Of use to us here is a set of concepts that has already had a role to play in ecological psychology – the distinction between thing and medium, as introduced by Heider (1959, originally published in German in 1926). In this important paper, Heider distinguishes between things whose components have relatively fixed relationships and a rigid structure, and media, whose components have only contingent or “spurious” relationships, making the whole fluid, and therefore tending to become rearranged by the structure of things moving through it. What happens in a medium depends on what impinge upon it. What happens to an object depends more on the existing relationships within it (Heider, 1959). The sense of medium then is not as channel of information, but fluid substrate which can come to be affected or formed by things, their structures and motions, thus allowing things to move through it, but also the structure of those things to propagate and impinge in various ways on other things, at a distance.

Heft (2001) explores the significant impact that Heider’s work had on Gibson’s conceptualization of perception, bringing into focus as it did not just the things to be perceived, but the means by which that perception could take place. The medium for Gibson is distinct from both substances (essentially, things) and surfaces (the planes of interface between things and medium). For terrestrial animals the medium of perception and locomotion is air, which is transparent and fluid. It supports the formation of ecological information in arrays of ambient light and thus direct perception.

Heider goes on to point out, however, that all media are made of things. So long as there is flexibility in their inter-relationships any sufficiently large aggregate of things can act as a medium. Building on Heider’s work Schoggen (1989) notes that the more things, the more flexible the medium. A Lego brick, for instance, is a rather rigid thing. A collection of 50 Lego bricks can be a medium for a variety of structures, and a collection of 500 Lego bricks even more so. The difference between a thing and medium is not absolute. This gives rise to the possibility of a nested set of media, in which the dynamics of things at one level of description act as an emergent medium at another, with each level of description having a particular set of characteristic dynamics – possibilities and constraints emerging from how the phenomena at that level of description operate.

As an illustration of how this might work we can look at how Heider’s ideas influenced another kind of ecological psychology – that of Roger Barker and colleagues, and also discussed in some depth by Heft (2007). The ideas of Barker and the work of the “Midwest Psychological Field Station” developed over years of observation of human behavior in the wild (or as wild as a small Rockwellesque town in rural Kansas in the 1950s and 60s was likely to get). As regards their different uses of Heider’s ideas, Heft (2001 p. 281) suggests Gibson’s is a *within-level* theory, examining relationships within a single animal-environment system, with Barker’s a *between-level* theory, concerned with how higher order structures in the social world come to shape the behavior of individuals.

It is beyond the scope of this paper to properly introduce the rich theory of person-environment interaction developed by Barker (1968) and Schoggen (1989) for full presentations, and Heft (2001) for an excellent introduction. Suffice it to note that for Barker et al., it is *people and their behavior* that are the medium, into which the standing patterns of organized social activity in appropriate places can impose their structure. These standing patterns of activity, with their accompanying physical milieu, are termed “behavior settings.”

Eventually situating his work somewhere between what we would normally call psychology, and sociology or anthropology, Barker adopted the term “eco-behavioral science” (Barker, 1978). He and his colleagues examined the ecosystem of human behavior at scales greater than individual actors or tasks. Barker warned that psychology as a discipline too frequently stepped outside of its competence (examination of the individual agent), and was too often asked to because there exists no theoretical framework for dealing with the factors that shape human behavior beyond the context of the immediate task, but beneath the broad domain of sociopolitical factors. The world is not randomly or probabilistically structured, and the transitions between one task and another do not occur in a stochastic manner for any given human being. In the vast majority of cases a person’s behavior coordinates very well with the setting in which they are working, and the sequences of settings that they experience from one end of the day to the other is neither accidental nor random. If we want to understand the structures of human behavior, he argued, we will need better theories of the structure of behavior settings, both as individual settings (usually involving multiple participants), and the relationships between settings, in buildings, neighborhoods, and cities. As Heft (2001, p. 258–259) points out, these structures are themselves made stable by sociopolitical forces and traditions. While Barker set his approach apart from sociological and anthropological concerns, it is vital to understand the ways in which power relations of gender, race, class, and other higher order dynamics play a role in the emergence of behavior settings. Heft (2001, p. 260) describes Barker as “offering a pluralistic perspective in the sense of requiring psychologists be sensitive to processes operating simultaneously at more than one level of analysis.” In this paper, I am simply amplifying or extending Barker’s approach. The possibility of multiple, nested, emergent levels of description, with “things” at one level of description having dynamics that allow them to operate as a medium for things at another level of description. This inter-level influence is not only one-way, however, as emergent systems also entrain, and therefore constrain, the dynamics of systems from which they emerge.

We must bear in mind that while a medium must have a significant malleability to allow itself to be shaped by things within it, no medium is perfect. As well as its own dynamics (some versions of tides or currents as we have already noted), any medium we identify will have certain structures that it can support, and others it cannot. These may be subtle, or they may not be (it is a version of this insight that led McLuhan, 1994 to utter his famous dictum that “the medium is the message”). In recent work, for instance, van Dijk and Kiverstein (2020) have explored the idea of a sociocultural practice as a medium of perception and action. Their analysis is broadly consistent with the approach I am advocating here, and they note the manner in which a medium can both enable and constrain the dynamics that emerge within it. Though they primarily deploy the concept of medium in a manner consistent with Gibson (a “within-level” analysis as Heft would have it), their work illustrates the way in which different levels of analysis interact, and in which things and media are not entirely independent.

Additionally, there is also no escaping our place as observers of these various emergent systems and their backgrounds. Fluidity at one temporal scale will look like rigid fixedness at another. We must be cautious, therefore, not to be too exclusionary in our descriptions of media and things at levels of description beyond those in which we ourselves most comfortably perceive and act, at least initially, and bear in mind our own perspective as scientists as playing a substantial role (Pask, 1996; Von Foerster, 2003).

I do not suggest that cognitive scientists somehow bear all levels of analysis in mind at any given time (I don’t consider it possible, though never say never, I suppose). What is more, I would not be confident that there is a single map of all of the various levels of analysis possible and proper to cognitive science is achievable, given the dynamism of the territory. It is quite likely that there are several sufficiently stable relationships between different forms of agency for us to explore which will give us some insight into what dimensions matter.

Barker’s approach explicitly acknowledges inter-level influence – tides and turbulence, as it were, for behavior. Analysis of complex systems offers us some tools for conceptualizing and systematically analyzing mutual influence and elasticity of relationships between levels of description, dynamics that may be invariant across scales where things become medium and enable the existence of new things. At any given level of analysis we can seek to identify and characterize the medium in question, and look for those aspects of it that can be ordered by the level of interest (what allows it to act as a medium), and those that impose themselves on the level of interest (what its limits as a medium are). This analysis can be done without taking the particular perspective of agent or environment in the approach, and may in time come to support the systematic conceptualisation of various flows of value independently of any perspective of a given biological agent embedded within the various processes of its environment.

## Conclusion: Cartographers Needed

Cognitive science has largely worked within a complex field built on methodologies and disciplinary traditions rather than an over-arching theoretical framework of how different forms of agency arise and interact at varied levels of analysis. There is certainly value to a pluralistic approach to understanding the mind, and some are quite fatalistic about such an over-arching framework (Gentner, 2019). I have suggested in this paper, though, that a substantial part of the apparent gap or miscoordination between ecological and enactive approaches has been a failure to recognize, and fully theorize this range of scales or levels of analysis, and how to systematically account for real differences between them. Though I don’t imagine the task will be simple, or perhaps ever completed, I think there remains value in an attempt to catalog what kinds of scale or dimensions matter, and to build a map with which we can situate any one program of research within the broader, complex territory in a principled manner. There will always be ambiguities and tensions between different domains of a science – the boundaries between biochemistry, genomics, morphology, and ecology, for instance, are occasionally contested, as are the relationships between them. But biology is the better for being able to orient research questions within these and other subfields in a way that, if not wholly coherent, is at least stable enough to support clear communication. The same is not currently possible within the cognitive sciences.

I have suggested that the complementary criticisms that have been leveled by enactivists and ecological psychologists against one another suggest that a new mode of description is warranted, one that can potentially avoid fracturing agent-environment descriptions from one another, while supporting a description of emergent dynamics. Such an ecumenical mode of description may support us adjudicating between such disputes, or diagnose them as like arguments over pronunciation by two groups of speakers with different accents. We are some ways away from an over-arching theoretical framework that integrates the two approaches within a fuller understanding of mind and world. If we can maintain an appreciation of their mutual dependence at all levels of description of the phenomena in question, we might be optimistic the some such framework is at least possible.

## Author Contributions

The author confirms being the sole contributor of this work and has approved it for publication.

## Conflict of Interest

The author declares that the research was conducted in the absence of any commercial or financial relationships that could be construed as a potential conflict of interest.

## Footnotes

1 Ecological psychologists most frequently refer to the “animal” or “organism”, in keeping with their strong emphasis on the concrete and particular. Fultot et al. (2016) have noted, however, that the principles of the ecological approach are sufficiently general as to apply regardless of whether or not the agent in question is living in this paper I have the for most part kept with the more generic and ecumenical “agent”.

## References

[B1] Baggs E. Chemero A. (2020). “The third sense of environment,” in *Perception as Information Detection: Reflections on Gibson’s Ecological Approach*, eds Wagman J. B. Blau J. J. C. (London: Routledge), 5–20. 10.4324/9780429316128-2

[B2] Barandiaran X. Di Paolo E. Rohde M. (2009). Defining agency:individuality, normativity, asymmetry and spatio-temporality in action. *Adapt. Behav.* 17 1–13.

[B3] Barker R. G. (1968). *Ecological Psychology: Concepts and Methods for Studying the Environment of Human Behavior.* Palo Alto, CA: Stanford University Press.

[B4] Barker R. G. (1978). “The need for an eco-behavioral science,” in *Habitats, Environments, and Human Behavior*, eds Roger G. Barker & Associates (San Francisco, CA: Jossey-Bass Publishers), 36–48.

[B5] Beaton M. (2016). Sensorimotor direct realism: how we enact our world. *Constr. Found.* 11 265–276.

[B6] Bender A. Hutchins E. Medin D. (2010). Anthropology in cognitive science. *Top. Cogn. Sci.* 2 374–385. 10.1111/j.1756-8765.2010.01082.x 25163866

[B7] Boden M. A. (2006). *Mind as Machine: A History of Cognitive Science.* Oxford: Oxford University Press.

[B8] Bruineberg J. Chemero A. Rietveld E. (2019). General ecological information supports engagement with affordances for ‘higher’ cognition. *Synthese* 196 5231–5251. 10.1007/s11229-018-1716-9 31814651 PMC6874517

[B9] Buhrmann T. Di Paolo E. A. Barandiaran X. (2013). A dynamical systems account of sensorimotor contingencies. *Front. Psychol.* 4:285. 10.3389/fpsyg.2013.00285 23750143 PMC3664438

[B10] Chemero A. (2009). *Radical Embodied Cognitive Science.* Cambridge, MA: MIT Press.

[B11] Costall A. (1995). Socializing affordances. *Theory Psychol.* 5 467–481. 10.1177/0959354395054001

[B12] Cuffari E. C. Di Paolo E. De Jaegher H. (2015). From participatory sense-making to language: there and back again. *Phenomenol. Cogn. Sci.* 14 1089–1125. 10.1007/s11097-014-9404-9

[B13] Cummins F. (2018). *The Ground from Which We Speak.* Cambridge, MA: Cambridge Scholars.

[B14] De Jaegher H. Di Paolo E. (2007). Participatory sense-making: an enactive approach to social cognition. *Phenomenol. Cogn. Sci.* 6 485–507. 10.1007/s11097-007-9076-9

[B15] De Jaegher H. Froese T. (2009). On the role of social interaction in individual agency. *Adapt. Behav.* 17 444–460. 10.1177/1059712309343822

[B16] Di Paolo E. (2005). Autopoiesis, adaptivity, teleology, agency. *Phenomenol. Cogn. Sci.* 4 429–452. 10.1007/s11097-005-9002-y

[B17] Di Paolo E. (2009). Extended life. *Topoi* 28 9–21.

[B18] Di Paolo E. (2016). Across the uncanny valley: the ecological, the enactive, and the strangely familiar. *Constr. Found.* 11 327–329.

[B19] Di Paolo E. (2018). “The enactive conception of life,” in *Oxford Handbook of Embodied, Embedded, Extended, Enactive Cognitive Science*, eds Newen A. De Bruin L. Gallagher S. (Oxford: Oxford University Press).

[B20] Di Paolo E. Barandiaran X. Buhrmann T. (2017). *Sensorimotor Life: An Enactive Proposal.* Oxford: Oxford University Press.

[B21] Di Paolo E. Cuffari E. C. De Jaegher H. (2018). *Linguistic Bodies.* Cambridge, MA: MIT Press.

[B22] Di Paolo E. A. Rohde M. De Jaegher H. (2010). “Horizons for the enactive mind: values, social interaction and play,” in *Enaction: Towards a New Paradigm of Cognitive Science*, eds Stewart J. Gapenne O. Di Paolo E. (Cambridge, MA: MIT Press).

[B23] Froese T. González-Grandón X. (2020). How passive is passive listening? Toward a sensorimotor theory of auditory perception. *Phenomenol. Cogn. Sci.* 19, 619–651. 10.1007/s11097-019-09641-6

[B24] Fultot M. Nie L. Carello C. (2016). Perception-action mutuality obviates mental construction. *Constr. Found.* 11 298–307.

[B25] Gentner D. (2019). Cognitive science is and should be pluralistic. *Top. Cogn. Sci.* 11 884–891. 10.1111/tops.12459 31564062

[B26] Gibson J. J. (1986). *The Ecological Approach to Visual Perception.* London: Psychology Press.

[B27] Gibson J. J. Gibson E. J. (1955). Perceptual learning: differentiation or enrichment? *Psychol. Rev.* 62:32. 10.1037/h0048826 14357525

[B28] Heft H. (2001). *Ecological Psychology in Context: James Gibson, Roger Barker, and the Legacy of William James’s Radical Empiricism*, 1st Edn New York, NY: Lawrence Erlbaum Associates.

[B29] Heft H. (2007). The social constitution of perceiver-environment reciprocity. *Ecol. Psychol.* 19 85–105. 10.1080/10407410701331934

[B30] Heider F. (1959). Thing and medium. On perception and event structure, and the psychological environment, Psychological issues, 1. *Monograph* 3 1–34.

[B31] Ignatow G. (2014). Ontology and method in cognitive science. *Sociol. Forum* 29 990–994. 10.1111/socf.12131

[B32] Kyselo M. (2014). The body social: an enactive approach to the self. *Front. Cogn. Sci.* 5:986. 10.3389/fpsyg.2014.00986 25309471 PMC4162380

[B33] Lobo L. Heras-Escribano M. Travieso D. (2018). The history and philosophy of ecological psychology. *Front. Psychol.* 9:2228. 10.3389/fpsyg.2018.02228 30555368 PMC6280920

[B34] Maturana H. R. Varela F. J. (1987). *The Tree of Knowledge: The Biological Roots of Human Understanding.* New Delhi: New Science Library.

[B35] McGann M. (2010). Perceptual modalities:Modes of presentation or modes of interaction? *J. Conscious. Stud.* 17 72–94.

[B36] McGann M. (2014). Enacting a social ecology: radically embodied intersubjectivity. *Cogn. Sci.* 5:1321. 10.3389/fpsyg.2014.01321 25477844 PMC4235264

[B37] McGann M. De Jaegher H. (2009). Self–other contingencies: enacting social perception. *Phenomenol. Cogn. Sci.* 8 417–437. 10.1007/s11097-009-9141-7

[B38] McGann M. Speelman C. (2020). Two kinds of theory: what psychology can learn from Einstein. *Theory Psychol.* 10.31234/osf.io/sp94q

[B39] McLuhan M. (1994). *Understanding Media: The Extensions of Man.* Cambridge, MA: MIT press.

[B40] Núñez R. Allen M. Gao R. Miller Rigoli C. Relaford-Doyle J. Semenuks A. (2019). What happened to cognitive science?. *Nat. Hum. Behav.* 3 782–791. 10.1038/s41562-019-0626-2 31182794

[B41] Pask G. (1996). Heinz von Foerster’s self-organization: the progenitor of conversation and interaction theories. *Syst. Res.* 13 349–363.

[B42] Rietveld E. Kiverstein J. (2014). A rich landscape of affordances. *Ecol. Psychol.* 26 325–352. 10.1080/10407413.2014.958035

[B43] Schoggen P. (1989). *Behavior settings: A revision and extension of Roger G. Barker’s “Ecological Psychology.”.* Palo Alto, CA: Stanford University Press.

[B44] Stoffregen T. A. (2003). Affordances as properties of the animal-environment system. *Ecol. Psychol.* 15:115 10.1207/S15326969ECO1502_2

[B45] Stoffregen T. A. Bardy B. G. (2001). On specification and the senses. *Behav. Brain Sci.* 24 195–213. 10.1017/S0140525X01003946 11530542

[B46] Stoffregen T. A. Mantel B. Bardy B. G. (2017). The senses considered as one perceptual system. *Ecol. Psychol.* 29 165–197. 10.1080/10407413.2017.1331116

[B47] Thompson E. (2005). Sensorimotor subjectivity and the enactive approach to experience. *Phenomenol. Cogn. Sci.* 4 407–427. 10.1007/s11097-005-9003-x

[B48] Thompson E. (2007). *Mind in Life: Biology, Phenomenology and the Sciences of Mind*, 1st Edn Cambridge, MA: Harvard University Press.

[B49] Torrance S. Froese T. (2011). An inter-enactive approach to agency: participatory sense-making, dynamics, and sociality. *Humana. Mente* 15 21–53.

[B50] Turvey M. T. Shaw R. E. Reed E. S. Mace W. M. (1981). Ecological laws of perceiving and acting: in reply to fodor and pylyshyn (1981). *Cognition* 9 237–304. 10.1016/0010-0277(81)90002-0 7197604

[B51] van Dijk L. Kiverstein J. (2020). Direct perception in context: radical empiricist reflections on the medium. *Synthese* 10.1007/s11229-020-02578-3

[B52] van Dijk L. Rietveld E. (2017). Foregrounding sociomaterial practice in our understanding of affordances: the skilled intentionality framework. *Front. Psychol.* 7:1969. 10.3389/fpsyg.2016.01969 28119638 PMC5220071

[B53] van Dijk L. Withagen R. (2016). Temporalizing agency: moving beyond on- and offline cognition. *Theory Psychol.* 26 5–26. 10.1177/0959354315596080

[B54] Varela F. J. (1997). Patterns of life: intertwining identity and cognition. *Brain Cogn.* 34 72–87. 10.1006/brcg.1997.0907 9209756

[B55] Varela F. J. Thompson E. Rosch E. (1991). *The Embodied Mind.* Cambridge, MA: MIT Press.

[B56] Von Foerster H. (2003). *Cybernetics of Cybernetics in Understanding Understanding.* Cham: Springer, 283–286.

[B57] Weber A. Varela F. J. (2002). Life after kant: natural purposes and the autopoietic foundations of biological individuality. *Phenomenol. Cogn. Sci.* 1 97–125. 10.1023/A:1020368120174

